# Exosomes derived from miR-126-3p-overexpressing synovial fibroblasts suppress chondrocyte inflammation and cartilage degradation in a rat model of osteoarthritis

**DOI:** 10.1038/s41420-021-00418-y

**Published:** 2021-02-24

**Authors:** Yan Zhou, Jianghua Ming, Yaming Li, Bochun Li, Ming Deng, Yonggang Ma, Zhonghui Chen, Yubiao Zhang, Jia Li, Shiqing Liu

**Affiliations:** 1 Department of Orthopedics, Renmin Hospital of Wuhan University, Wuhan, China; 2 Central Laboratory, Renmin Hospital of Wuhan University, Wuhan, China; 3 Department of Acupuncture, Wuhan Union Hospital, Huazhong University of Science and Technology, Wuhan, China; 4 College of Acupuncture and Bone Injury, Hubei University of Traditional Chinese Medicine, Wuhan, China

**Keywords:** Drug development, Apoptosis

## Abstract

MicroRNAs (miRNAs) encapsulated within exosomes can serve as essential regulators of intercellular communication and represent promising biomarkers of several aging-associated disorders. However, the relationship between exosomal miRNAs and osteoarthritis (OA)-related chondrocytes and synovial fibroblasts (SFCs) remain to be clarified. Herein, we profiled synovial fluid-derived exosomal miRNAs and explored the effects of exosomal miRNAs derived from SFCs on chondrocyte inflammation, proliferation, and survival, and further assessed their impact on cartilage degeneration in a surgically-induced rat OA model. We identified 19 miRNAs within synovial fluid-derived exosomes that were differentially expressed when comparing OA and control patients. We then employed a microarray-based approach to confirm that exosomal miRNA-126-3p expression was significantly reduced in OA patient-derived synovial fluid exosomes. At a functional level, miRNA-126-3p mimic treatment was sufficient to promote rat chondrocyte migration and proliferation while also suppressing apoptosis and IL-1β, IL-6, and TNF-α expression. SFC-miRNA-126-3p-Exos were able to suppress apoptotic cell death and associated inflammation in chondrocytes. Our in vivo results revealed that rat SFC-derived exosomal miRNA-126-3p was sufficient to suppress the formation of osteophytes, prevent cartilage degeneration, and exert anti-apoptotic and anti-inflammatory effects on articular cartilage. Overall, our findings indicate that SFC exosome‐delivered miRNA-126-3p can constrain chondrocyte inflammation and cartilage degeneration. As such, SFC-miRNA-126-3p-Exos may be of therapeutic value for the treatment of patients suffering from OA.

## Introduction

Osteoarthritis (OA) is an arthritic disease that is driven by synovial inflammation, progressive cartilage degradation, osteophyte formation, and subchondral bone remodeling, ultimately adversely impacting quality of life as affected individuals age^1,2^. Acute synovitis is among the first joint-related changes to appear in those suffering from OA, and synovial tissues of patients with early-stage OA exhibit elevated pro-inflammatory mediator levels^3,4^. As such, synovial fluid samples can be analyzed in order to monitor and study pathophysiological alterations affecting the joints, articular cartilage, and related tissues. Synovial fibroblasts (SFCs) secrete this synovial fluid, which in turn lubricates the articular cartilage^5,6^. Elevated levels of pro-inflammatory factors such as interleukin-1β (IL-1β), tumor necrosis factor (TNF), and nitric oxide (NO) within the synovial fluid can promote osteophyte formation and cartilage lesion development^7^. Inflammatory cytokines (such as TNF, IL-1β, IL-6, and certain chemokines) can alter chondrocyte differentiation, function, and viability, while also inducing the activation and expression of cartilage-degrading matrix metalloproteinases (MMPs) and aggrecanases that drive OA pathogenesis^8,9^. Anti-inflammatory approaches to treating synovitis thus represent a powerful means of preventing or slowing the progression of OA.

Exosomes and other forms of extracellular vesicles have been found to serve as carriers of macromolecules including proteins, mRNAs, and microRNAs (miRNAs)^10,11^. Exosome-derived proteins and miRNAs can alter the survival and differentiation of cells into which they are internalized, and are thus likely to influence the development and progression of OA^12,13^. Exosomes are secreted by myriad tissues and cell types, and SFC- and neutrophil-derived exosomes have been detected in the synovial fluid of OA patients^14^. By mediating pro-inflammatory intercellular communication, these exosomes can promote angiogenesis, the degradation of the extracellular matrix, and antigen presentation^15,16^. Positive feedback signaling between SFCs and chondrocytes in the joint can further exacerbate local inflammation, with exosomes, in particular, serving as key mediators of this process owing to their ability to transmit inflammatory proteins and miRNAs to both proximal and distal tissues^17^.

The targeting of miRNAs capable of regulating chondrocyte-specific gene expression represents a potentially attractive approach to treating OA^18–20^. While miRNAs within the synovial fluid are generally rapidly degraded, exosomes can stabilize these molecules and thereby improve their ability to alter proliferation, differentiation, survival, and inflammatory activity in other cells^21^. Previous analyses of synovial fluid-derived exosomes from OA patients have found that samples from these patients exhibit a 2.5-fold increase in miR-200-c levels relative to control patient samples. This miRNA was able to suppress zinc-binding transcription factor expression and thereby disrupt the secretion of type II collagen^22^. Exosomes containing pro-chondrogenic miRNAs would theoretically be capable of replacing damaged chondrocytes in the context of OA^23^. As such, additional studies of how specific miRNAs control chondrogenesis may lead to the development of potent regenerative therapeutic tools capable of suppressing OA progression or reversing disease-related damage.

Owing to the high levels of inflammatory proteins and exosomes found in synovial fluid, the synovial secretome is an important determinant of synovial morphology and disease-related pathology in those with OA^24^. By understanding the miRNAs present within synovial exosomes in OA patients, it may be possible to better understand the mechanistic basis for this complex disease and to identify novel therapeutic targets amenable to treatment in affected individuals. As such, we herein isolated synovial fluid exosomes from patients with and without OA and employed a microarray-based approach to identify exosomal miRNAs that were differentially expressed between these two patient populations. Of these, miR-126 was studied in-depth as it is an important transcriptional regulator of certain inflammation-related mediators^25^. We ultimately determined that miR-126-3p was downregulated by 2.93-fold in synovial exosomes from OA patients. Reduced miR-126 expression led to the decreased stability of the cell-matrix attachment network, consistent with the tissue lesions observed in OA^26^. Moreover, miR-126 has been suggested to play a role in cell aging and senescence^27,28^ and may be associated with cartilage homeostasis and OA. However, little evidence exists regarding the role of miR-126 in joint pathology and OA. In this study, we explored the functional relevance of exosomal miR-126-3p in order to better understand its role as a suppressor of SFC-mediated chondrocyte inflammation and cartilage degradation. Overall, our data highlight exosomes as novel therapeutic tools or targets for the treatment of OA.

## Materials and methods

### Patient sample collection

**Fig. 1:**
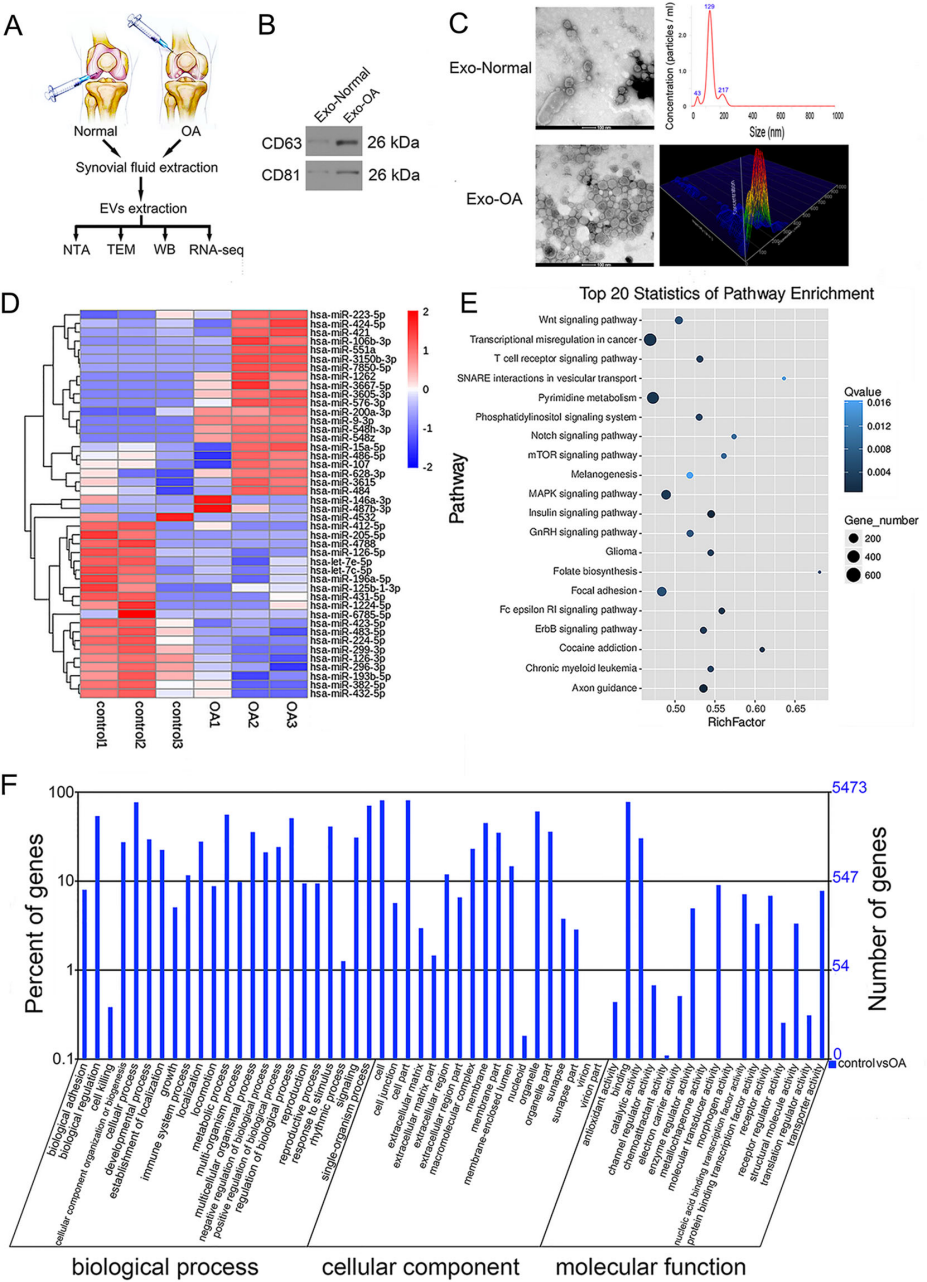
Exosomes derived from human synovial fluid and bioinformatics analysis for exosome-encapsulated miRNAs. **A** The synovial fluid of NON-OA and OA patients was obtained. Then the extracellular vesicles obtained by ultrafiltration assay were collected and analyzed. **B** The indicated protein levels (CD63 and CD81) in exosome-like vesicles were detected by western blotting. **C** The morphology of exosome-like vesicles was observed under TEM and NanoSight detection was measured. **D** Heatmap label for aberrantly expressed miRNAs in synovial fluid exosomes of patients with OA. **E**, **F** The KEGG pathway annotation and GO analysis were performed.

Samples of synovial fluid from ten OA patients (male, five, female, five; age 62.6 ± 6.3 years) undergoing total knee replacement surgery were collected, as were synovial fluid samples from ten patients (male, six, female, four; age 58.5 ± 7.1 years) without OA or rheumatoid arthritis who were undergoing arthrocentesis procedures. All synovial fluid samples were collected during surgery, after which they were immediately taken to a laboratory in order to isolate the exosomes therein (Fig. 1A). All samples were collected at Renmin Hospital of Wuhan University from January 2019 to December 2019. The present study was approved by the ethics committee of Renmin Hospital of Wuhan University (2019K-K011). All of the participants in the present study provided written informed consent to the ethics committees of Renmin Hospital of Wuhan University.

### Exosome-like vesicle isolation and assessment

An ultracentrifugation approach was used to isolate exosomes. Briefly, samples of synovial fluid (Fig. 1A) and SFC supernatants (Fig. 3A) were subjected to centrifugation at 300 × *g* (10 min), and the pellet was removed (Fig. 3A). Then the supernatant was again spun at 2000 × *g* (10 min) and 10,000 × *g* (30 min) at 4 °C. Subsequently, supernatants were spun for 70 min at 100,000 × *g* in an SW28 rotor (Beckman Coulter, Brea, CA) in order to pellet exosomes. The pellet was then resuspended in phosphate-buffered saline (PBS) and spun down for 70 min at 100,000 × *g* at 4 °C. The collected exosome-like vesicles were analyzed via transmission electron microscopy (TEM; FEI TECNAI G2, USA) and nanoparticle tracking analyses (Malvern Nanosight NS300, UK).

### Exosome miRNA isolation and microarray profiling

A miRNeasy Kit (Qiagen, Hilden, Germany) was used to isolate miRNAs from exosomes based on provided directions, after which miRNA quantity and quality were assessed using a NanoDrop spectrophotometer (Thermo Scientific, Waltham, MA) and an Agilent Technologies 2100 Bioanalyzer (Agilent Technologies, USA). An Affymetrix GeneChip® miRNA 4.0 Array (Affymetrix, USA) was then used to profile exosome-derived miRNAs. A hierarchical clustering approach was then used to generate heat maps comparing miRNA expression profiles between OA and control samples. The data were extracted from scanned images using the Feature Extraction software v.10.7 (Agilent Technologies, USA) and normalized using the Gene Spring Software v.11.0 (Agilent Technologies).

### Analysis of differential miRNA expression and function

PCR data were analyzed using a web-based PCR array data analysis software (http://pcrdataanalysis.sabiosciences.com/pcr/arrayanalysis.php). The miRNA PCR array data in the OA and control groups were initially normalized using a global normalization method^29^, after which miRNAs that were differentially expressed between these two groups (OA and NON-OA) were identified via *t*-tests using the following cutoff criteria: *P* < 0.05, fold-change >1.5. To minimize the potential noise introduced by measurements below the detection threshold, miRNAs, with Ct values greater than 35 in OA and NON-OA groups were considered to be undetected. Gene ontology (GO) and Kyoto encyclopedia of genes and genomes (KEGG) enrichment analyses of these differentially expressed miRNAs were then conducted with DIANA-miRPath v 3.0 (http://diana.imis.athena-innovation.gr/DianaTools/index.php), and the Wordle software (www.wordle.net) was used to generate corresponding word clouds. In addition, principal component analyses were conducted.

### Cell culture and transfection

Knee joints were collected from Sprague-Dawley (SD) rats, after which enzymatic digestion was employed to isolate primary rat SFCs and articular chondrocytes. Animals were obtained from the Center for Animal Experiment/ABSL-III Laboratory of Wuhan University (Wuhan, China). Briefly, samples of cartilage and synovial tissue were minced into 0.5–1 mm^3^ fragment prior to treatment for 1 h with 0.25% Trypsin and 0.02% EDTA. Samples were then treated for an additional 4 h with 0.2% type II collagenase at 37 °C, after which collected cells were resuspended in complete DMEM/F12 containing 10% fetal bovine serum and cultured at 37 °C in a humidified 5% CO_2_ incubator. For transfection, cells were resuspended in complete DMEM/F12 and plated for 24 h until 70–80% confluent, at which time they were transfected with 100 pM of miR-126-3p mimic or inhibitor (RiboBio, Guangzhou, China) constructs based on provided directions. All protocols were approved by the Institutional Ethics Committee of Medical School, Wuhan University.

### Chondrocyte proliferation and colony formation assay

A Cell Counting Kit-8 (CCK-8; Dojindo, Kumamoto, Japan) was used to gauge the impact of miR-126-3p or SFC-derived miR-126-3p‐containing exosomes (SFC-miR-126-3p-Exos) on rat chondrocyte proliferation. Briefly, chondrocytes from the third round of passage were added to 96-well plates (0.5 × 10^4^/well) for 12 h, after which they were treated for 2 h with a range of miR-126-3p (100 pM) or SFC-miR-126-3p-Exo (2 × 10^9^/mL) concentrations before 0.75 mM sodium nitroprusside (SNP) (Youcare Pharmaceutical Group Co., Ltd, Beijing) treatment was conducted for 24–48 h. At appropriate time points, 100 μl of 10% CCK-8 solution was added per well for 2 h, after which a microplate reader (Bio-Tek, Model EXL800, USA) was used to assess absorbance at 450 nm. Colony formation assays were also used to assess the impact of miRNA-126-3p mimics (100 pM) or inhibitors (100 pM) on chondrocyte proliferation. Briefly, 500 cells were added to 25 cm^2^ culture dishes for 24 h after which they were cultured for 2 weeks. Following a PBS wash, these cells were then fixed for 20 min with 10% formalin and stained for 1 h using 0.5% crystal violet (Sigma). Images were then scanned with an Olympus microscope (Olympus Corporation, Tokyo, Japan), and total colony numbers were counted.

### Cell cycle analysis

Chondrocytes were added to complete media in the presence of miR-126-3p mimic (100 pM) or inhibitor (100 pM) constructs for 24 h. Cells were then harvested, fixed using 70% ethanol, and stored for 24 h at 20 °C. Cells were then washed twice using PBS and were treated for 30 min with a solution supplemented with 25 μg/ml Ribonuclease A and 50 μg/ml propidium iodide (MultiSciences Biotech Co., Ltd., Hangzhou) based on provided directions. A FACScan flow cytometer (Becton Dickinson, USA) was then used to analyze samples.

### Assessment of apoptosis

An Annexin V-FITC/PI kit (MultiSciences Biotech Co., Ltd., Hangzhou, China) was used to assess rates of chondrocyte apoptosis based on provided directions. Briefly, chondrocytes were spun down for 5 min at 1000 rpm, washed twice using cold PBS, and resuspended in 500 µl of binding buffer containing 5 µl each of PI and Annexin V-FITC. Following a 15 min incubation protected from light, cells were assessed using a FACScan flow cytometer (Becton-Dickinson, USA) in order to quantify apoptotic rates.

### Quantitative real-time polymerase chain reaction (qRT-PCR)

Trizol (Carlsbad, CA, USA) was used to extract total RNA from miRNA- or exosome-treated chondrocytes based on provided directions. Absorbance at 260 nm and 280 nm was then used to assess RNA quantity and purity, after which a PrimeScript RT Reagent kit (Dalian, China) was used to prepare cDNA. SYBR Premix Ex TaqII (TaKaRa, Dalian, China) was then used together with an Eco Real-Time PCR System (Illumina, Shanghai, China) for all qRT-PCR reactions. GAPDH was used as a normalization control, while the 2^(−ΔΔCT)^ method was used to assess relative gene expression. Primers used for this study are shown in Table 1.

**Table 1:** Primers of targeted genes.

Gene	Forward	Reverse
IL-1β	5′–GTGGCAGCTACCTATGTCTTGC–3′	5′–CCACTTGTTGGCTTATGTTCTGT–3′
IL-6	5′–GCCAGAGTCATTCAGAGCAAT–3′	5′–CTTGGTCCTTAGCCACTCCT–3′
TNF-α	5′–CACCACGCTCTTCTGTCTACTG–3′	5′–GCTACGGGCTTGTCACTCG–3′
GAPDH	5′–GCCAAGGTCATCCATGACAAC–3′	5′–GTGGATGCAGGGATGATGTTC–3′

### Western blotting

RIPA buffer supplemented with a protease inhibitor cocktail (Sigma) was used to lyse extracellular vesicles, after which a Bradford assay (Bio-Rad Laboratories) was used to measure total protein levels in these samples. Blots were then probed overnight using antibodies specific for CD9 (1:1000, Abcam), CD63 (1:1000, Abcam), CD81 (1:1000, Abcam), HSP70 (1:2000, Abcam), IL-1β (1:500, Bioss), IL-6 (1:1000, Abcam), and TNF-α (1:500, Abcam) at 4 °C. Secondary HRP-conjugated goat anti-rabbit IgG antibody (1:10000, ASPEN) was then used to probe these blots, after which an ECL Western blot detection system (Thermo Scientific, Waltham, MA) was used to detect protein bands.

### Animal studies

The animal experiments were carried out according to the recommendations in the Guide for the Animal Care and Use Committee of Medical School, Wuhan University (WDRM 20160104). The functional impact of SFC-miR-126-3p-Exos on the articular cartilage was assessed in 30 male SD rats (200–250 g; from the Center for Animal Experiment/ABSL-III Laboratory of Wuhan University, Wuhan, China). These rats were then randomized into a sham operation group, an SFC-miRNA-126-3p-Exos group, an OA-induction group, an OA + SFC-control-Exos group, and an OA + SFC-miRNA-126-3p-Exos group. All animals were anesthetized via the intraperitoneal injection of trichloroacetaldehyde hydrate (300 mg/kg body weight) in saline solution. An OA model was then established by transecting the anterior cruciate ligament and resecting the medial menisci (ACLT + MMx) in the right knee based on provided directions^30^. At 1 week postoperatively, rats were transferred into a rotating electric cage for 30 min per day as in our prior study^31^. At 4 weeks post-surgery, rats in the appropriate treatment groups were intra-articularly injected with 40 μl of 500 μg/ml SFC-miRNA-126-3p-Exos or SFC-control-Exos once per week. Animals in the sham operation group were injected once per week with 40 μl of PBS in the right knee joint. At 10 weeks post-surgery, all rats were euthanized via cardiac exsanguination. Micro-MRI, micro-CT, histology, TUNEL assay, and immunohistochemistry were used as evaluation criteria for study animals (Fig. 4A).

### In vivo micro-MRI

An in vivo 9.4 T high‐field micro-MRI (BioSpec 70/30 USR, Germany) approach was used to evaluate the right knee joints of each rat. The MRI was operated in a fat‐suppressed, spin‐echo, T2‐weighted scan mode in order to visualize potential bone marrow lesion-like/edema‐like phenomena within tissue samples. Sagittal medial femoral condyle sections from each dataset were selected, and the ImageJ software (NIH Image, National Institutes of Health, Bethesda, MD; online at: http://rsbweb.nih.gov/ij/) was then used to quantify epiphyseal water signals therein. Mean visual pixel intensity within a circular 1.8 mm diameter region of interest selected based upon the presence of the metaphyseal growth plate and the cortical bone margins were evaluated by a single investigator.

### Micro-CT analysis

A micro-CT instrument (Skyscan 1276, Bruker microCT N.V., Kontich, Belgium; 4000 × 2672 pixels, 9 μm isotropic voxel size) was used to assess rat knee joints. Collected images were then assessed based on overall structural appearance, as well as on quantitative morphometric indices (bone volume fraction [BV/TV, %], average trabecular thickness [Tb. Th, mm], and average trabecular separation [Tb. Sp, mm]) measured using 3D morphometric micro-tomographic data.

### Histological analyses

Following rat knee joint disarticulation, tibial plateau and femoral condyle samples were fixed for 24 h with 4% PFA. Calci-Clear Slow solution [10% (w/v) EDTA, pH 7.4] was then used to treat samples for 3 weeks in order to achieve decalcification, after which samples were paraffin-embedded. Next, 5 mm serial sagittal cartilaginous tissue sections were prepared and used for hematoxylin and eosin (H&E) staining.

### TUNEL assay

Chondrocyte cell death was evaluated using an in situ cell death detection kit (KeyGEN Biotech, Nanjing, China) based upon the presence of DNA fragmentation. Briefly, sagittal cartilaginous tissue sections from knee joints were treated with 20 μg/ml proteinase K (Dako, Glostrup, Denmark) for 15 min, after which apoptotic chondrocytes in the articular cartilage were labeled. Numbers of apoptotic cells were then quantified in five randomly selected high-power fields (×100) of view per sample in tissues from each group.

### Immunohistochemical staining

Levels of IL-1β (1:200, Bioss) and TNF-α (1:200, Abcam) in cartilaginous tissues were analyzed via immunohistochemistry. Briefly, serial sagittal cartilaginous tissue sections were probed overnight at 4 °C with appropriate primary antibodies. Primary antibody binding in these sections was then detected using a diaminobenzidine staining kit (Boster Biological Engineering, Wuhan, China), after which hematoxylin was used to counterstain samples. Optical microscopy was then used to image these cells, and Image-Pro Plus 6.0 (Media Cybernetics Co., USA) was used to quantify staining results based upon integrated optical density.

### Statistical analysis

Data are means ± standard error of the mean (SEM), and were compared via one-way ANOVAs or Student’s *t*-tests as appropriate using SPSS 13.0 or GraphPad Prism 5.0 (San Diego, CA, USA). *P* < 0.05 was the significance threshold for this study.

## Results

### Synovial fluid samples from humans contain exosomes

We began by collecting synovial fluid samples from OA or control patients, after which a total exosome isolation reagent was used to collect exosomal particles from these samples. When these collected exosomes were assessed via Western blotting, they were found to be positive for the exosomal markers CD63 and CD81 (Fig. 1B). These exosomes exhibited a generally round morphology when evaluated via TEM (Fig. 1C), and were roughly 100 ± 10 nm in diameter when assessed via nanoparticle tracking analysis.

### OA alters the expression of miRNAs within synovial exosomes

The overall concentrations and morphology of synovial fluid exosomes were comparable in samples from OA and control patients. In contrast, we found that miRNA concentrations differed significantly when comparing exosomes from these two patient groups (Table 2). In total, 12 and 7 miRNAs were upregulated and downregulated in OA patient synovial exosomes relative to control patient synovial exosomes, respectively. In a heat map constructed via a supervised clustering approach, clear differences were evident between OA and control patient samples. These findings thus indicated that OA pathology is associated with marked changes in miRNA expression profiles within synovial fluid exosomes (Fig. 1D). Of the miRNAs that were differentially abundant between these two patient groups, we found that exosomal miR-126-3p levels were reduced 2.93-fold in the synovial exosomes from OA patients (OA-Exos) relative to those from control patients (NON-OA‐Exos).

**Table 2:** Selected miRNAs differentially regulated in OA patients and normal controls.

miRNA	Fold change	*P*-value	Regulation
hsa-miR-423-5p	−2.488214054	0.040698815	Down
hsa-miR-126-3p	−2.930388381	0.027642982	Down
hsa-miR-486-5p	3.442247864	0.0162373	Up
hsa-miR-382-5p	−3.305739887	0.017134542	Down
hsa-miR-196a-5p	−2.609695276	0.045405736	Down
hsa-miR-126-5p	−3.475883077	0.01164154	Down
hsa-miR-432-5p	−2.91444871	0.043808753	Down
hsa-miR-107	2.648370383	0.043865305	Up
hsa-miR-15a-5p	2.760703304	0.038934669	Up
hsa-miR-21-5p	−0.968104319	0.487175733	Down
hsa-miR-16-5p	1.500799548	0.375680983	Up
hsa-miR-199a-3p	−1.140187751	0.307854149	Down
hsa-miR-199b-3p	−1.139867316	0.307904921	Down
hsa-miR-146a-5p	0.848484628	0.411926506	Up
hsa-miR-148a-3p	−0.493260217	0.589913859	Down
hsa-miR-29a-3p	−0.147949043	0.969506163	Down
hsa-miR-125b-5p	0.742710167	0.51408735	Up
hsa-miR-221-3p	0.476254006	0.799929679	Up
hsa-miR-122-5p	−0.648616457	0.416793116	Down

### Functional enrichment analyses of differentially expressed miRNAs

To understand the putative functional roles of miRNAs that were differentially expressed when comparing OA-Exos and NON-OA-Exos, we conducted GO and KEGG functional enrichment analyses. KEGG pathway analyses revealed these miRNAs to be significantly linked to pathways including proliferation, migration, metabolism, and signal transduction (Fig. 1E). These pathways included the “transcriptional misregulation in cancer”, “pyrimidine metabolism”, “folate biosynthesis”, “MAPK signaling”, “Wnt signaling”, “phosphatidylinositol signaling system”, “T cell receptor signaling”, “axon guidance”, “insulin signaling”, and “mTOR signaling” pathways. In total, 1671 targets of these miRNAs were identified and found to be enriched in 23, 18, and 17 GO biological process, cellular component, and molecular function terms, respectively (Fig. 1F).

### MiR-126-3p promotes chondrocyte proliferation and suppresses apoptosis and inflammation

**Fig. 2:**
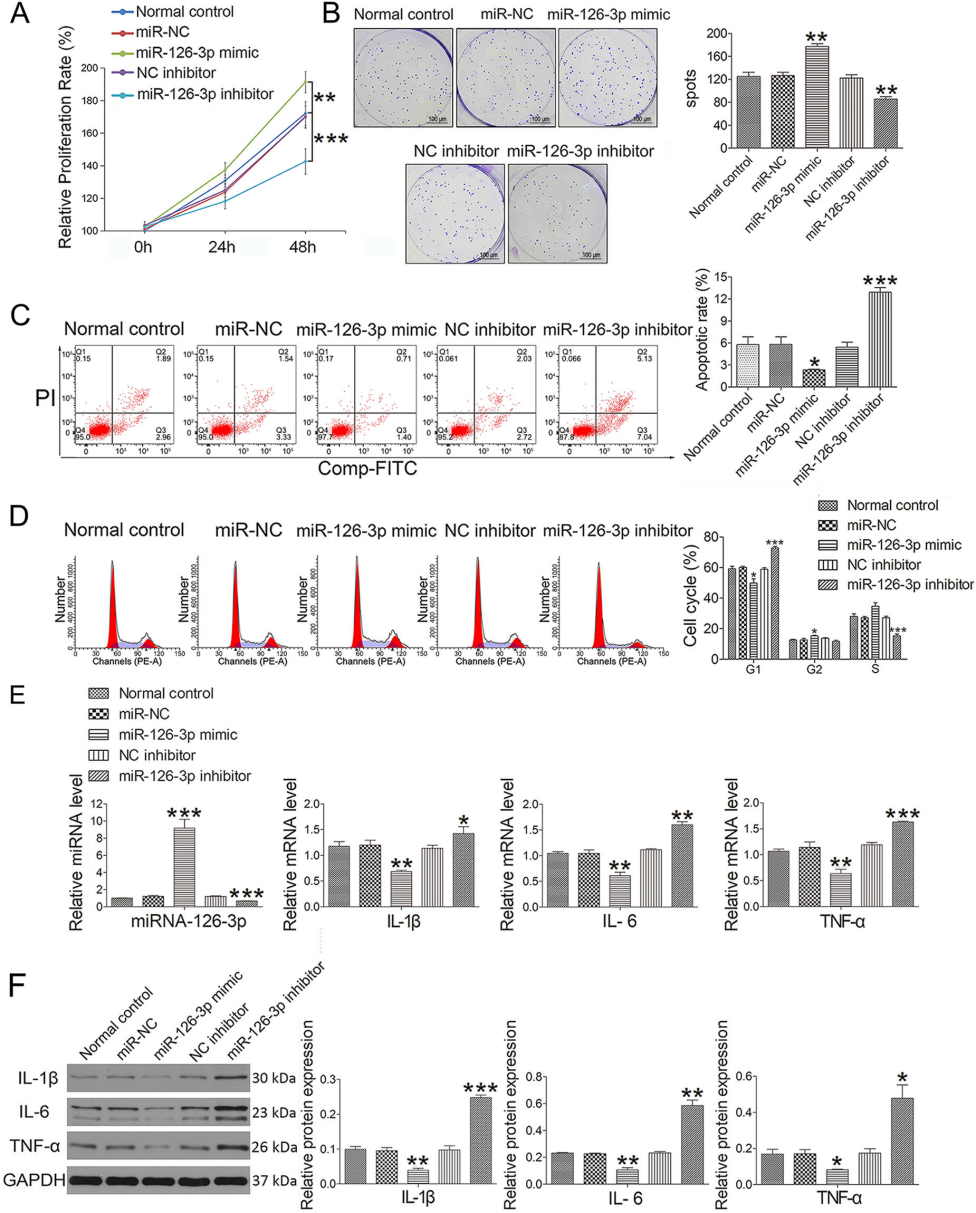
Effect of miR-126-3p administration on chondrocytes. **A** Cell proliferation was determined by CCK-8 at concentrations of 100 pM (miRNA-126-3p mimic and inhibitor) for 24 and 48 h. **B** The colony formation assay was used to analyze the effect of miRNA-126-3p mimic (100 pM) and miRNA-126-3p inhibitor (100 pM) on the proliferation of chondrocytes. **C** Apoptotic index was determined using flow cytometry in the presence of miR-126-3p mimic (100 pM) or inhibitor (100 pM) constructs for 24 h. **D** Effect of miR-126-3p on cell cycle progression in the presence of miR-126-3p mimic (100 pM) or inhibitor (100 pM) constructs for 24 h. **E** The miRNA (IL-1β, IL-6, and TNF-α) and mRNA (miRNA-126-3p) expression was detected by qRT-PCR in the presence of miR-126-3p mimic (100 pM) or inhibitor (100 pM) constructs for 24 h. **F** Western blot assay was used to detect inflammation-related proteins (IL-1β, IL-6, and TNF-α) in the presence of miR-126-3p mimic (100 pM) or inhibitor (100 pM) constructs for 24 h. Data were expressed as mean ± SEM (*n* = 3). ^*^P < 0.05, ^**^P < 0.01, and ^***^P < 0.001 vs. normal control.

As the levels of miR-126-3p were significantly reduced in exosomes derived from OA patients, we hypothesized that this miRNA may be associated with OA pathogenesis. A CCK8 analysis revealed that chondrocytes had been transfected with miR-126-3p mimics exhibited increased proliferation relative to control cells, whereas the opposite phenotype was observed following miR-126-3p inhibitor transfection (Fig. 2A). To further explore the impact of this miRNA on the growth of chondrocytes, a colony formation assay was conducted. Whereas the colony formation of miR-126-3p mimic-transfected chondrocytes was enhanced relative to control cells, chondrocytes that had been transfected with miR-126-3p inhibitors exhibited impaired colony formation (Fig. 2B). An Annexin V-FITC/PI dual staining approach was additionally used to evaluate the impact of this miRNA on chondrocyte apoptosis. Following miR-126-3p mimic or inhibitor transfection, 2.33 ± 0.21% and 12.94 ± 1.08% of chondrocytes were found to be apoptotic, respectively, with both of these percentages differing significantly relative to the control group (Fig. 2C). We further found that there were significantly fewer chondrocytes in the G1 cell cycle phase and significantly more chondrocytes in the G2 phase following miR-126-3p transfection (Fig. 2D). In contrast, miR-126-3p inhibitor transfection was associated with a reduction in the frequency of chondrocytes in the S phase and an increase in the frequency of chondrocytes in the G1 phase. Protein and mRNA levels of IL-1β, IL-6, and TNF-α were also significantly decreased in the miR-126-3p mimic treatment group and enhanced in the miR-126-3p inhibitor treatment groups (Fig. 2E, F).

### SFC-miR-126-3p-Exos suppress chondrocyte inflammation and apoptosis

**Fig. 3:**
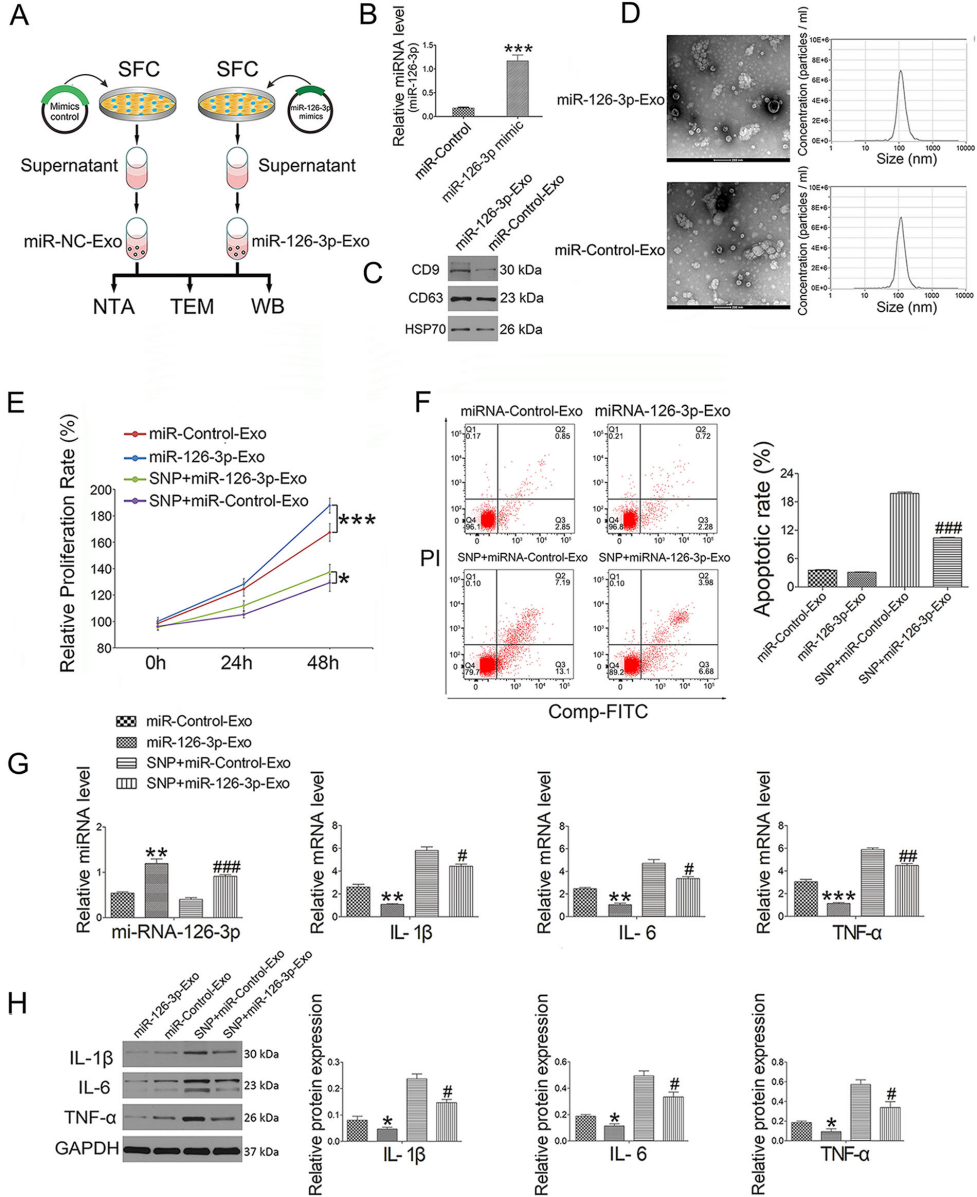
Separation and identification of SFC-derived exosome-like vesicles and the effect of SFC-miR-126-3p-Exos administration on chondrocytes. **A** Rat SFC was isolated and cultured in cell culture media. Then the supernatant exosome-like vesicles extracted using ultracentrifugation assay were subjected to NanoSight detection. **B** The miRNA expression (miR-126-3p) of SFC transfected with miR-126-3p mimic (100 pM) was detected by qRT-PCR for 24 h incubation. **C** The indicated protein levels (CD9, CD63, and HSP70) in exosome-like vesicles were detected by western blotting. **D** The morphology of exosome-like vesicles was observed under TEM and NanoSight detection was measured. **E** Cell proliferation treated by SFC-miR-126-3p-Exos was determined by CCK-8 at concentrations of 2 × 10^9^/mL for 24 and 48 h. **F** Apoptotic index was determined using flow cytometry in the presence of SFC-miR-126-3p-Exos (2 × 10^9^/mL) for 2 h before 0.75 mM SNP co-treatment for 24 h. In this study, 0.75 mM fresh SNP was used to induce chondrocyte inflammation. **G** The miRNA (miR-126-3p) and mRNA expression (IL-1β, IL-6, and TNF-α) were detected by qRT-PCR in the presence of SFC-miR-126-3p-Exos (2 × 10^9^/mL) for 2 h before 0.75 mM SNP co-treatment for 24 h. **H** Western blot assay was used to detect inflammation-related (IL-1β, IL-6, and TNF-α) proteins in the presence of SFC-miR-126-3p-Exos (2 × 10^9^/mL) for 2 h before 0.75 mM SNP co-treatment for 24 h. Data were expressed as mean ± SEM (*n* = 3). ^*^P < 0.05, ^**^P < 0.01, and ^***^P < 0.001 vs. miR-Control-Exo; ^#^*P* < 0.05, ^##^*P* < 0.01, and ^###^*P* < 0.001 vs. SNP + miR-Control-Exo.

We next assessed the morphology of SFC-miR-126-3p-Exos, revealing these particles to be ~100 ± 10 nm in diameter, consistent with the expected characteristics of exosomes. The miR-126-3p mimic could successfully transfect SFCs, showing a high expression of miR-126-3p (Fig. 3B). Exosomal marker proteins (CD9, CD63, and HSP70) were also expressed by these exosomes, confirming the identity of these collected SFC-miR-126-3p-Exos (Fig. 3C). These exosomes also exhibited a dual-layer membrane morphology and a cup-like shape (Fig. 3D). A CCK8 analysis revealed that the treatment of chondrocytes with these SFC-miR-126-3p-Exos enhanced their proliferation in the presence or absence of SNP relative to control cells (Fig. 3E). Chondrocytes that had been treated with SNP and SFC-miR-126-3p-Exos exhibited an apoptotic frequency of 10.37 ± 0.27%, which was significantly decreased relative to that observed in chondrocytes that had been treated with SNP and control SFC-derived exosomes not overexpressing miR-126-3p (SFC-miR-control-Exos) (19.78 ± 0.46%) (Fig. 3F). We further confirmed that SFC-miR-126-3p-Exo treatment was associated with a significant increase in miR-126-3p expression, and with decreases in IL-1β, IL-6, and TNF-α expression at the mRNA (Fig. 3G) and protein levels (Fig. 3H) as compared to SFC-miR-control-Exo-treated cells.

### In vivo micro-MRI analysis results

**Fig. 4:**
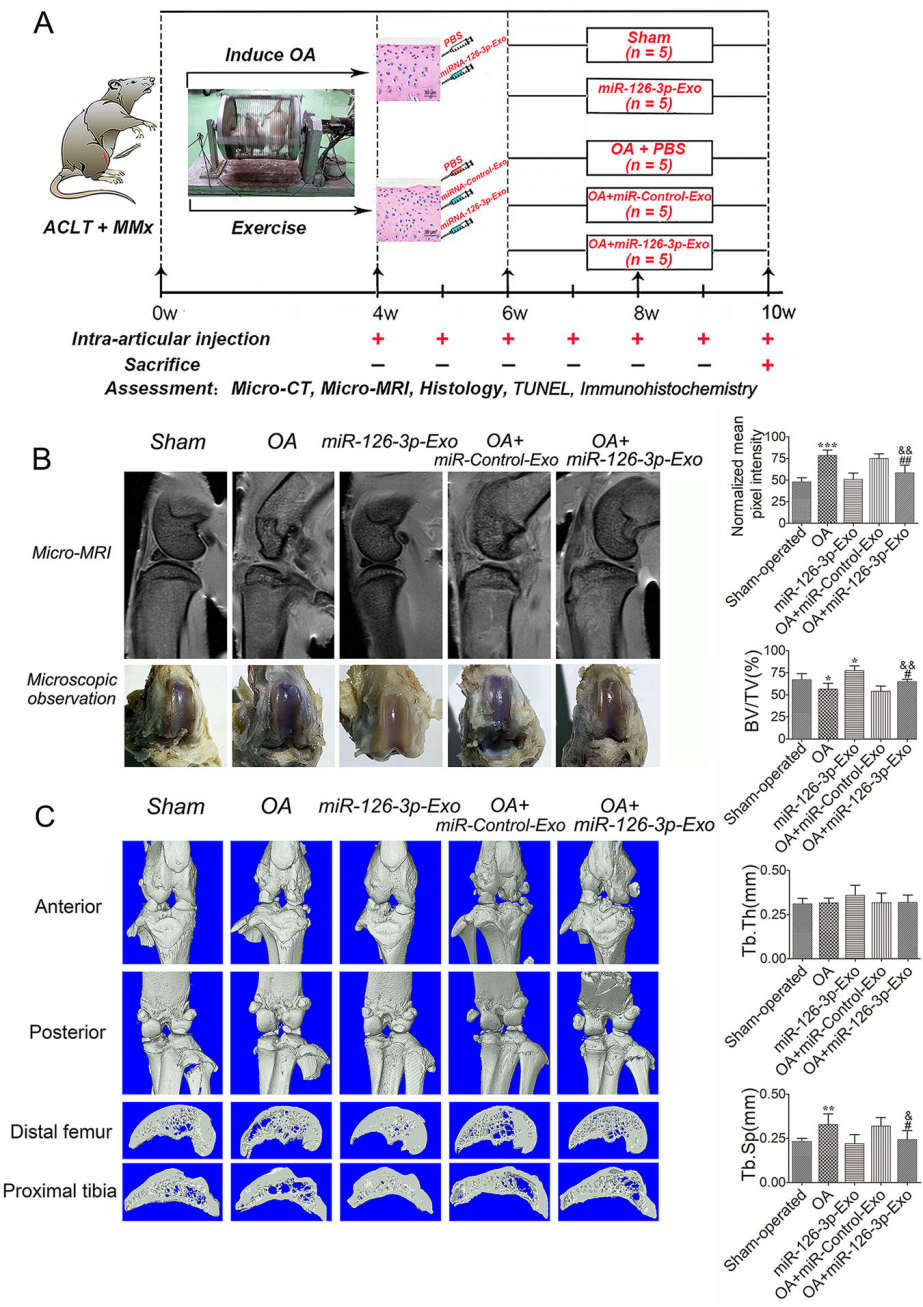
An overview of study timelines and the rescue of cartilage degeneration by SFC-miR-126-3p-Exos in OA model rats. **A** ACLT + MMx rats were put into an electronic rotator cage for 30 min per day as a means of inducing the OA model beginning 1-week post-surgery. At 4 weeks post-surgery, animals were intra-articularly injected with 40 μl of 500 μg/ml SFC-miR-126-3p-Exos. PBS was used as a control in sham and OA-model animals. At 10 weeks post-surgery, the micro-MRI, micro-CT, histology, TUNEL assay, and immunohistochemistry were used as evaluation criteria. **B** Pixel intensity of a sampled region of epiphyseal trabecular bone and gross morphological were assessed by micro-MRI to quantify the presence of bone marrow lesions in sham operation, SFC-miRNA-126-3p-Exos, OA-induction, OA + SFC-control-Exos, and OA + SFC-miRNA-126-3p-Exos, groups. **C** Representative micro-CT three-dimensional reconstructions of tibial and femoral subchondral bones in the rats after ACLT + MMx surgery. Micro-CT images of animals in each group were used for measurements of BV/TV, Tb. Th and Tb. Sp. Data were expressed as mean ± SEM (*n* = 5). ^*^P < 0.05, ^**^P < 0.01, and ^***^P < 0.001 vs. sham-operated group; ^#^*P* < 0.05 and ^##^*P* < 0.01 vs. OA induction group; ^&^*P* < 0.05 and ^&&^*P* < 0.01 vs. OA + miR-Control-Exos group.

We next employed a micro-MRI approach to assessing the T2 signal in the epiphyseal subchondral bone marrow of rats in our OA model group (Fig. 4B). Measurements of this epiphyseal bone compartment were made based upon the use of the endosteal bone envelope as an anatomic landmark. While fat‐suppressed, T2‐weighted micro‐MRI images from sham-operated control rats exhibited a clear, thin T2 signal consistent with the metaphyseal growth plate, rats in the OA model group exhibited significantly increased T2 signal in this location consistent with abnormal lesion- or edema-like inflammation within the bone marrow. The T2 signal intensity was significantly reduced in OA model rats that had been treated with SFC-miR-126-3p-Exos relative to untreated OA model controls, whereas there was no significant difference in the T2 signal between sham-operated rats and SFC-miRNA-126-3p-Exo-treated rats.

### SFC-miR-126-3p-Exos maintain subchondral bone structure in a rat model of OA

For this study, we leveraged an ACLT + MMx rat model of OA, with micro-CT being used to assess changes in the cartilage and subchondral bone including altered calcification, joint space morphology, and osteophyte formation (Fig. 4C). Compared to sham-operated control rats, those in the OA model group exhibited reductions in the BV/TV ratio and increases in Tb. Sp. These changes were reversed, however, when rats were treated via the intra-articular administration of SFC-miR-126-3p-Exos.

### SFC-miR-126-3p-Exos suppress synovial inflammation-mediated cartilage degeneration in OA model rats

**Fig. 5:**
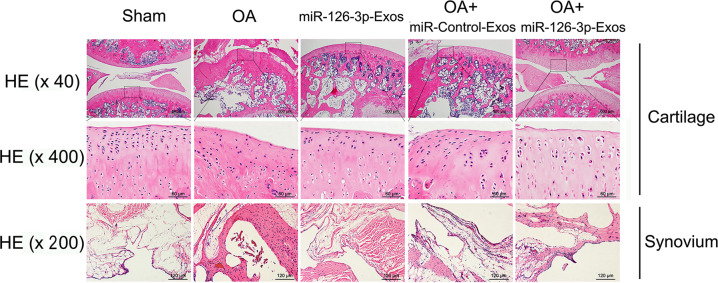
SFC-miR-126-3p-Exos suppress synovial inflammation-mediated cartilage degeneration. Sections of articular cartilage and synovium (*n* = 5 for each group) were stained using H&E staining. Those OA model rats treated with SFC-miRNA-126-3p-Exos exhibited increased surface regularity and articular cartilage thickness, and alleviate synovial inflammation.

H&E staining was used to assess changes in histology in OA model rats (Fig. 5). Relative to sham-operated control animals, those in the OA model group exhibited irregular articular cartilage morphology, whereas those OA model rats treated with SFC-miRNA-126-3p-Exos exhibited increased surface regularity and articular cartilage thickness consistent with less severe cartilage degradation. Synovial thickening was evident in OA model rats and was significantly reduced in OA + SFC-miRNA-126-3p-Exo-treated animals, consistent with the ability of these SFC-miRNA-126-3p-Exos to alleviate synovial inflammation.

### SFC-miR-126-3p-Exos inhibited the apoptotic death and inflammation of articular cartilage chondrocytes in a rat model of OA

**Fig. 6:**
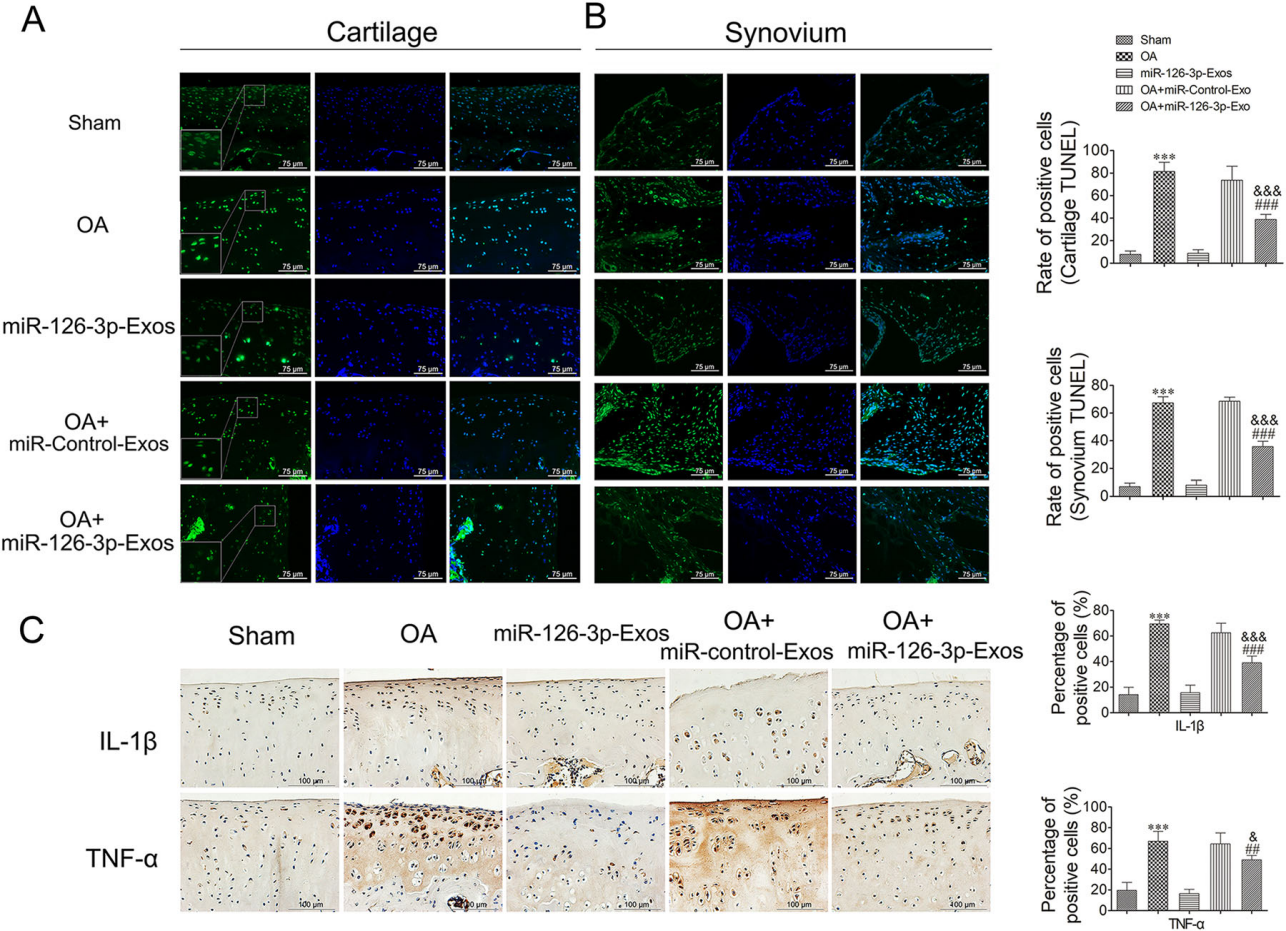
Assessment of apoptosis and inflammation in cartilage mediated by SFC-miR-126-3p-Exos in OA model rats. **A** At 10 weeks post-surgery, animals were sacrificed and the knee joints were collected for TUNEL analysis. A representative TUNEL-stained section of articular cartilage was used to assess apoptosis. **B** Representative TUNEL-stained section of synovial tissues was used to assess apoptosis. **C** The immunohistochemical stainings of IL-1β and TNF-α were performed in OA model rats. The ratios of immunoreactive positive cells were analyzed. Data were expressed as mean ± SEM (*n* = 5). Data were expressed as mean ± SEM (*n* = 5). ^***^P < 0.001 vs. sham-operated group; ^##^*P* < 0.01 and ^###^*P* < 0.001 vs. OA induction group; ^&^*P* < 0.05, ^&&^*P* < 0.01, and ^&&&^*P* < 0.001 vs. OA + miR-Control-Exos group.

The impact of SFC-miR-126-3p-Exo treatment on the apoptotic death of chondrocytes and SFCs in this rat model system was next assessed via TUNEL staining. Relative to sham-operated control rats, samples from OA model rats exhibited significantly higher frequencies of TUNEL-positive cells in the articular cartilage (Fig. 6A) and synovial tissues (Fig. 6B), whereas these frequencies were significantly reduced in OA model rats treated with SFC-miR-126-3p-Exos. Lastly, we employed an immunohistochemical staining approach to assess IL-1β and TNF-α expression in cartilage tissue sections from model rats. These experiments revealed that the IL-1β and TNF-α levels were markedly reduced in OA model rats treated with SFC-miRNA-126-3p-Exos relative to untreated OA model rats (Fig. 6C).

## Discussion

In the present study, we explored the ability of synovial fluid-derived exosomal miRNAs to modulate OA pathogenesis and knee joint degeneration. Using miRNA microarrays we identified many miRNAs that were differentially abundant in synovial exosomes from OA patients including upregulated miRNAs (miR-107, miR-15a-5p, miR-16-5p, miR-146a-5p, miR-125b-5p, and miR-221-3p) as well as downregulated miRNAs (miR-126-3p, miR-423-5p, miR-382-5p, miR-196a-5p, and miR-432-5p), suggesting that these factors may modulate cartilage pathophysiology. Notably, we determined that exosomal miR-126-3p was downregulated 2.93-fold in synovial fluid samples from OA patients relative to control patients, and we determined that this miRNA was associated with OA disease activity. Exosomes derived from SFCs overexpressing miR-126-3p were sufficient to suppress inflammation in rat articular chondrocytes and to delay OA progression.

Synovial fluid can be monitored to assess pathophysiological alterations in the knee joint space, as it is closely associated with the articular cartilage and the synovial membrane in this tissue site^32,33^. Cell-derived exosomes represent particularly promising tools for diagnostic biomarker identification when evaluating disease progression, as they can both reflect and influence pathophysiological conditions within a given microenvironmental setting^34,35^. Herein, we, therefore, conducted an analysis of miRNAs within exosomes derived from the synovial fluid of OA and control patients. These particles were confirmed to be morphologically consistent with exosomes (~100 ± 10 nm in diameter) and were present at similar levels in the synovial fluid of OA and control patients. In addition, these exosomes contained high levels of miRNAs.

To evaluate the functional relevance of miRNAs that were differentially expressed within synovial fluid exosomes from OA patients, we conducted GO and KEGG enrichment analyses of the target genes of these miRNAs. This approach revealed these exosomal miRNAs to be closely linked to key pathways associated with proliferation, migration, metabolism, and signal transduction. OA-related pathways identified via this approach included the MAPK, Wnt, mTOR, and phosphatidylinositol signaling pathways. We further found that synovial fluid-derived exosomes were readily endocytosed by chondrocytes, whereupon they were able to alter signaling processes within these cells. We, therefore, hypothesized that OA patient-derived synovial exosomes may impact metabolic activity within articular chondrocytes. Our data supported this hypothesis, as do prior findings from Kato et al., who determined that exosomes collected from IL-1β-stimulated human SFCs were able to promote enhanced MMP-13 and ADAMTS-5 expression and to suppress COL2A1 expression in articular chondrocytes when compared with exosomes from non-stimulated cells^36^. Our results showed that exosomes derived from SFCs overexpressing miR-126-3p were able to suppress chondrocyte inflammation more effectively than were exosomes derived from control SFCs, suggesting that these SFC-derived exosomes may be key regulators of synovial inflammatory responses.

There is substantial evidence that miRNAs can influence immune and inflammatory responses by altering the expression of genes associated with cellular activation, differentiation, and apoptosis^37^. We thus hypothesized that exosome-derived miRNAs from inflamed synovial tissues may control bone degeneration and related joint remodeling. As miR-126-3p has been shown to play important roles in the context of OA progression, we assessed the ability of exosome-derived miR-126-3p to influence the pathogenesis of OA. Our hypothesis was that this miRNA may be sufficient to inhibit inflammatory cytokine production following the SNP treatment of rat chondrocytes. SNP is a fast-acting vasodilator that is often used as an external NO donor in clinical and basic research^38^. Consistent with our hypothesized model, we found that proliferation and SNP‐induced IL-1β, IL-6, and TNF‐α production in chondrocytes were markedly suppressed following miR-126-3p overexpression, and miR-126-3p-overexpressing SFCs were able to effectively suppress OA progression.

We additionally evaluated the therapeutic utility of isolated SFC-miR-126-3p-Exos in the treatment of ACLT + MMx-induced OA model rats. Intra-articular injection of exosomes can effectively promote cartilage tissue regeneration and prevent OA progression^39,40^. We found that administering these exosomes into the articular cartilage was sufficient to suppress OA progression and to enhance the regeneration of cartilaginous tissue, preventing the development of severe damage in these model rats. Importantly, these SFC-miR-126-3p-Exos exhibited superior therapeutic efficacy as compared to control exosomes such that OA-related proteoglycan loss, irregular surface morphology, synovial inflammation, and superficial fibrillation were no longer evident following SFC-miR-126-3p-Exo treatment, whereas they were still detectable after control exosome treatment. In this rat OA model, the bone marrow lesion-like phenomenon identified in micro-MRI analyses can be used as a measure of OA disease progression and drug efficacy. Bone marrow lesions are characterized by an increase in signal in fat suppression T2-weighted scans^41^. Micro-CT and micro-MRI analyses demonstrated that SFC-miR-126-3p-Exo treatment was associated with significantly higher bone volume fraction values and with a reduction in lesion-like/edema-like inflammatory abnormalities in the bone marrow relative to control exosome treatment. These findings led us to conclude that SFC-miR-126-3p-Exo treatment was sufficient to promote healing in this rat model of OA.

While our results provided clear evidence that exosomal miR-126-3p can effectively suppress the degeneration of cartilaginous tissues, there are nonetheless certain limitations to this study. Our sample size was limited. Future studies of more OA patients with varying levels of disease severity will be essential. At present, we are planning experiments to explore signaling pathway activity in human chondrocytes in an effort to more fully understand the complexities of the regulation of chondrogenesis.

**Fig. 7:**
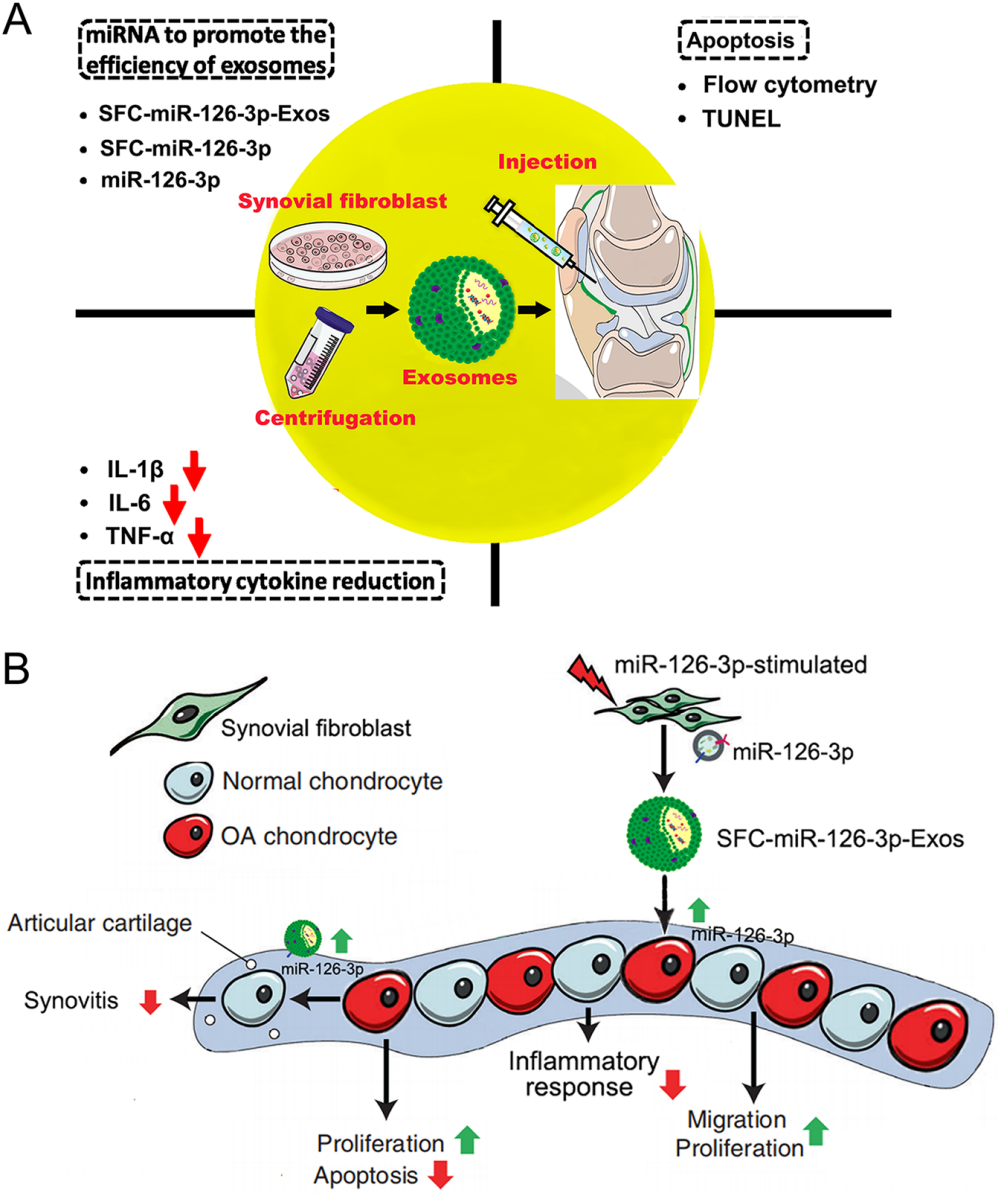
Proposed mechanism of SFC-miR-126-3p-Exos interference with OA chondrocyte inflammation and cartilage degradation. **A** The therapeutic effects of SFC-derived exosomes on OA are mainly reflected in the following three parameters. (1) reduction of inflammatory cytokines, (2) inhibition of apoptosis, and (3) modulate miRNA-126-3p in exosome to promote the therapeutic efficiency. **B** The exosomes derived from SFC could mediate cell–cell communications and regulate diverse cell phenotype including inflammatory reaction, cell proliferation, migration, apoptosis and etc.

In summary, our findings indicate that exosome-derived miRNAs are key regulators of OA pathology. SFC exosome‐delivered miR-126-3p can drive anti-inflammatory signaling, ultimately suppressing proinflammatory cytokine production and OA progression (Fig. 7).
